# Unsupervised Learning and Pattern Recognition of Biological Data Structures with Density Functional Theory and Machine Learning

**DOI:** 10.1038/s41598-017-18931-5

**Published:** 2018-01-11

**Authors:** Chien-Chang Chen, Hung-Hui Juan, Meng-Yuan Tsai, Henry Horng-Shing Lu

**Affiliations:** 1 Bio-Microsystems Integration Laboratory, Department of Biomedical Sciences and Engineering, National Central University, Taoyuan City, Taiwan; 2 Shing-Tung Yau Center, National Chiao Tung University, 1001 University Road, Hsinchu City, Taiwan; 3 Institute of Statistics, National Chiao Tung University, 1001 University Road, Hsinchu City, Taiwan; 4 Big Data Research Center, National Chiao Tung University, 1001 University Road, Hsinchu City, Taiwan

## Abstract

By introducing the methods of machine learning into the density functional theory, we made a detour for the construction of *the most probable* density function, which can be estimated by learning relevant features from the system of interest. Using the properties of universal functional, the vital core of density functional theory, the *most probable* cluster numbers and the corresponding cluster boundaries in a studying system can be simultaneously and automatically determined and the plausibility is erected on the Hohenberg-Kohn theorems. For the method validation and pragmatic applications, interdisciplinary problems from physical to biological systems were enumerated. The amalgamation of uncharged atomic clusters validated the unsupervised searching process of the cluster numbers and the corresponding cluster boundaries were exhibited likewise. High accurate clustering results of the Fisher’s iris dataset showed the feasibility and the flexibility of the proposed scheme. Brain tumor detections from low-dimensional magnetic resonance imaging datasets and segmentations of high-dimensional neural network imageries in the *Brainbow* system were also used to inspect the method practicality. The experimental results exhibit the successful connection between the physical theory and the machine learning methods and will benefit the clinical diagnoses.

## Introduction

Due to multifarious data expansions that rapidly arise from user generated contents and log-data formed in internet surfing, social media^1–5^, investigations of pathology and DNA sequence^6,7^, bioscience of biological networks^2,8–11^, cloud and heterogeneous computing^12^, explosive growth of global financial information^13–15^, and so forth, relevant techniques in the field of *Big Data* are ambitiously developing under the considerations of commercial strategies and scientific investigations. Among these applications in the era, the methodologies of analyzing biological data structures especially attract wide attention both in industry and academia. For instance, the morphological visualizations of neural networks in human brains are worthy of attention due to the urge of seeking correspondences among the brain functionalities with physiological and psychological modulations, pathological diagnoses, perceptual characteristics, and the rest. Once a comprehensive map of neural circuitry in human brains, the so-called connectome^16^, can be clearly delineated and clarified, the scientists can deeply understand the basic functions within the human brains. In practice, a well-investigated morphology of cerebral neurons can benefit clinical diagnoses to detect the regions of neural miswiring connection related to Alzheimer’s and Parkinson’s diseases^17^. In the field of magnetic resonance image processing, state-of-the-art techniques successfully combined several merits from interdisciplinary methods. The imaging techniques of 3-dimensional morphologies associated with soft-clustering methods exhibit an opportunity to track the brain regions related to relevant diseases^18,19^.

However, the progress on connectomics staggered^17,20–22^. The reason can be attributed to the scarcity of robust and reliable automatic methods for neural tracing and segmentation, so that immense and tedious manual interventions became inevitable to deal with humongous and intricate neural networks. Until an exquisite transgenic strategy named a *Brainbow* system was proposed, the mentioned predicaments are alleviated. By introducing colored fluorescent proteins with stochastic combinatorial expressions into the neural networks^23,24^, the *Brainbow* system immediately made an avenue on the requirements of discriminating the large-scale neural circuitry using random colors.

Several relevant technical obstacles, however, hamper the way of analyzing color mappings of the large-scale neural networks. A dataset of *brainbow* images usually occupies a large memory size of several hundred mega-bytes and contains about 10^8^ data points in the pixel space^20,24–27^. Snaking neurons stitch confusedly intertwining patterns, thus the chrominance within an imaging voxel would be possibly contaminated by adjacent components. This severe color crosstalk tends to undesirably penalize spurious branches and premature terminations when the image resolution is compromised^24,25,28^, and then causes a fallible neural network tracing and segmentation. In addition, the saturation of fluorescence also results in localized luminance pollution within voxels. Saturated luminous intensity within the voxels can probably cause not only topological errors on neural clustering but also the bogus neural connectivity.

To circumvent these deficiencies, state-of-the-art techniques based on the machine learning in probabilistic perspectives have brought fruitful achievements^20,21,24,25,29–31^. By combining the graphic theory with geometric features^17,22,26–34^ and topological priors^20,24,25,34^ of neurons, morphologies of neural networks could be delineated visually. For fulfilling neural mapping decompositions, these machine learning-based algorithms rely on certain prerequisites, such as training sets^29^ and seeding voxels^20,24,25,34^, regular curves or shape of axons^17,22,27,28,31–34^, designated sizes^32^, and so forth. Among these investigations, the method of spectral matting^25^ provides a sequential searching by optimizing a Laplacian type cost function to extract neural components from different color channels. Thus, the segmentation problem becomes an optimization problem. In this method, the window size in the Laplacian cost function is a crucial parameter and should be defined in advance. Bayesian Sequential Partitioning algorithm^20^ for probabilistic modeling is also a feasible method for neural segmentation. The time complexity caused by the detection of the direction of voxel growth, however, is a challenging issue. Therefore, user-supervised interventions and the algorithm complexity would probably become inevitable in the irregular or unanticipated circumstances.

In order to erect unsupervised learning methods in the relevant applications, specific physical methods have also caught attentions from data scientists who are solving interdisciplinary problems in the field of data analysis. Due to the equivalence between the Markov random field in statistics and the Gibbs energy distribution in thermodynamics, the datasets in problems of interest can be analogized to a lattice-like physical system by linking the Bayesian posterior distribution and the specific energy function^35^. Thus, a physical system and energy states whereof have definitely one-to-one correspondences between random variables and the corresponding outcomes respectively. Ideally, features of a dataset can be mapped into corresponding physical states. Then the method of simulated annealing^35,36^ can be used to search the most possible state as well as the maximum a posteriori probability in the statistical learning. Once the prior information, observations, and an appropriate hypothesis for describing the intrinsic properties of a dataset are given, the Bayesian-based approaches are easy to implement on the problems of interest. If the prior information is missing or lacking, the method of the quantum clustering provides an alternative^37^. By constructing a time-independent Schrödinger wave function in the ground state using the Parzen probability distribution with one pending parameter, cluster centers of a dataset can be well determined by sequentially searching the minima of the potential function in the system. Then, the accuracy of the data clustering will depend on the determination of the mentioned pending parameter.

Because the pending parameter in an unknown system only could be measured by exhaustively searching its appropriate value, the time consuming process and the user intervention might possibly limit its applications in large-scale data structures. Therefore, to reinforce the performance from physical methods, we propose a new unsupervised learning methodology by combining the framework of the density functional theory and the methods in the field of machine learning. The density functional theory^38,39^, as a broad consensus, provides an elegant framework to handle the pragmatic problems in solid-state theory and quantum chemistry^40–45^. The physical configuration and characteristics within an *N*-particle system can be fully elucidated using a 3-dimensional electronic density^44–46^ rather than processing 3*N*-dimensional many-particle wave functions. In other words, the framework of the density functional theory provides the convenience of computational complexity reduction. Hence, its mathematical framework in these aspects reveals highly beneficial suitability and compatibility for investigating large-scale systems.

In this report, a connection between the density functional theory and the methods of machine learning was successfully constructed by mapping the information from the physical space to the data space. An unsupervised searching algorithm of the cluster number as well as the corresponding cluster boundaries was proposed based on the Hohenberg-Kohn theorems. The current study is the pioneering attempt to propose such a methodology to solve these issues. Several interdisciplinary problems of pattern recognition from physical to biological systems were enumerated to elucidate the feasibility and the accuracy of the proposed algorithm. Therefore, the proposed algorithm reveals a successful connection between the physical methods and the machine learning methods.

## Methods

The density functional theory exhibits an avenue for constructing a rigorous many-body theory by employing the electron density *ρ* as a fundamental quantity. As the theoretical vital core, the universal functional *F*[*ρ*], sum of the true kinetic energy and electron-electron interaction, is completely independent of any system so that it applies equally well from hydrogen atoms to the DNA. However, the explicit forms of true kinetic energy *T*[*ρ*] and electron-electron interaction *E*_*ee*_[*ρ*] are still completely in the dark. Approximations thus become inevitable in pragmatic applications.

Inspired by the field of machine learning, we made a detour for the formula construction of universal functional. The electron density under our proposed scheme might be measured using any sophisticated method in the field of machine learning. The effects due to the lack of explicit forms would be parameterized, and the utilized machine learning method will fulfill intricate estimations by learning relevant features from the system of interest. Differing from conventional parametric methods, such as the adiabatic connection^47,48^ or the mentioned quantum clustering, the proposed method provides an unsupervised learning approach so that the relevant features relied on the measured electron density would be automatically extracted and then analyzed.

The essence of an electron density is actually a probability density function (PDF). In a statistical perspective, a particle PDF of an *N*-body system can be expressed as a *conditional joint* PDF:

1 $$\rho {\boldsymbol{=}}\rho {\boldsymbol{(}}{{\boldsymbol{r}}}_{{\bf{1}}}{\boldsymbol{,}}{{\boldsymbol{r}}}_{{\bf{2}}}{\boldsymbol{,}}\ldots {\boldsymbol{,}}{{\boldsymbol{r}}}_{{\bf{N}}}{\boldsymbol{)}}{\boldsymbol{=}}\rho {\boldsymbol{(}}{\boldsymbol{R}}{\boldsymbol{|}}{\boldsymbol{w}}{\boldsymbol{,}}{\boldsymbol{X}}{\boldsymbol{,}}{\sum }^{{\bf{2}}}{\boldsymbol{)}}{\boldsymbol{,}}$$

where ***R*** = [***r***_1_, ***r***_2_, …, ***r***_*N*_]^***T***^, ***X***, and ***w*** are a feature matrix, an attribute matrix, and a coefficient matrix, respectively. In a physical space, a measure ***r***_*i*_ represents a three-dimensional position vector so that the feature matrix can be recognized as a coordinate matrix likewise at this moment. Σ^2^ is a systematic covariance and can model the noise and the statistical dependence between features. The attribute matrix ***X*** is composed of all observable vectors of particles, ***x***_1_, ***x***_2_, …, ***x***_*N*_, and has the form ${\boldsymbol{X}}={[{{\boldsymbol{x}}}_{1}^{T},{{\boldsymbol{x}}}_{2}^{T},\ldots ,{{\boldsymbol{x}}}_{N}^{T}]}^{T}$. Each observable vector ***x***_*i*_ includes corresponding observables of *i*th particle, such as its coordination number, Coulombic amount, and so forth. The coefficient matrix ***w*** includes weighting factors corresponding to each element of the observable vectors. These factors can be determined either by learning from the system or by governed scientific principles.

Also inspired by the concept of non-interacting reference system in the density function theory, the conditional joint PDF expressed in Eq. (1) can be simplified as a product of univariate Gaussian distributions ${\mathscr{N}}$:

2 $$\rho ({\boldsymbol{R}}|{\boldsymbol{w}},{\boldsymbol{X}},{{\rm{\Sigma }}}^{2})=\prod _{i=1}^{N}\rho ({{\boldsymbol{r}}}_{i}|{\boldsymbol{w}},{\boldsymbol{X}},{\sigma }^{2})=\prod _{i=1}^{N}{\mathscr{N}}({{\boldsymbol{w}}}^{T}{{\boldsymbol{x}}}_{i},{\sigma }^{2}),$$

where ***w***^*T*^***x***_*i*_ and *σ*^2^ are the mean and the variance for each corresponding Gaussian distribution. It is noted that the measures in Eq. (2) are now *conditionally independent* with each other and the original dependence is erected by the structure of the coefficient matrix ***w***^49^. In other words, the statistical dependence between these measures are conditionally bridled to the coefficient matrix. Therefore, the realistic and interacting system could now be treated as a non-interacting reference one. Those effects due to the lack of explicit functionals are immediately embedded into the coefficient matrix, which will be determined by learning from the system. In an ideal case, by estimating the maximum log-likelihood, the estimated coefficient matrix and variance can have close forms $\hat{{\boldsymbol{w}}}={({{\boldsymbol{X}}}^{T}{\boldsymbol{X}})}^{-1}{{\boldsymbol{X}}}^{T}{\boldsymbol{R}}$ and ${\hat{\sigma }}^{2}=[{{\boldsymbol{R}}}^{T}{\boldsymbol{R}}-{{\boldsymbol{R}}}^{T}{\boldsymbol{X}}\hat{{\boldsymbol{w}}}]/N$, respectively. Then the systematic uncertainty can be elaborated using the Fisher information matrix in terms of the attribute matrix and the variance. In a pragmatic application, furthermore, the particle PDF under the Gaussian-based cluster estimate can be expressed as:

3 $$\rho ({\boldsymbol{R}}|{\boldsymbol{w}},{\boldsymbol{X}},{{\rm{\Sigma }}}^{2})=\sum _{j=1}^{M}\prod _{i=1}^{N}{\pi }_{j}{\mathscr{N}}({{\boldsymbol{w}}}_{j}^{T}{{\boldsymbol{x}}}_{ij},{{\rm{\Sigma }}}_{j}^{2}),$$

where *π*_*j*_, ***w***_*j*_ and Σ_*j*_ are the cluster weighting factor, the coefficient matrix, and the covariance of *j*th cluster of total *M* clusters, respectively. The ***x***_*ij*_ is *i*th observable vector pertaining to the *j*th cluster.

To construct the connection between an energy PDF and the particle PDF in the proposed scheme, the local density approximation^50,51^ was adopted to study the corresponding circumstances between the data length and the Fermi surface. The Fermi surface corresponds to an outermost surface of a system of interest in an energy space. At the moment, for a pragmatic transformation, a particle is now treated as a data point under the statistical perspective. First, the data length in a *D*-dimensional energy space is:

4 $$N=\frac{{{\mathscr{V}}}_{D}}{{(2\pi )}^{D}}{\int }_{0}^{{k}_{F}}dk\cdot {\alpha }_{D}{k}^{D-1}=\frac{{\alpha }_{D}{{\mathscr{V}}}_{D}{k}_{F}^{D}}{D{(2\pi )}^{D}},$$

where ${{\mathscr{V}}}_{D}$ and *α*_*D*_ are *D*-dimensional hyper-volume and dimension-dependent integral constant, respectively. Parameters *k* and *k*_*F*_ are magnitudes of the wave vector and the Fermi surface, respectively. Thus, the relation between the data PDF *ρ* and *k*_*F*_ can be obtained:

5 $$\rho =\frac{N}{{{\mathscr{V}}}_{D}}=\frac{{\alpha }_{D}{k}_{F}^{D}}{D{(2\pi )}^{D}}\,{\rm{or}}\,{k}_{F}[\rho ]=2\pi {[\frac{D}{{\alpha }_{D}}\cdot \rho ]}^{1/D}.$$

Obviously, the data PDF is a functional of *k*_*F*_, and vice versa. It is noted that the data PDF will be measured using the common machine learning methods as mentioned. Under the scheme of local density approximation with the sufficient boundary information from *k*_*F*_[*ρ*], the expectation of kinetic energy density functional (KEDF) can be derived as:

6 $$t[\rho ]=\frac{\frac{{{\mathscr{V}}}_{D}}{{(2\pi )}^{D}}{\int }_{0}^{{k}_{F}}dk\cdot {\alpha }_{D}{k}^{D-1}\cdot (\frac{{k}^{2}}{2})}{\frac{{{\mathscr{V}}}_{D}}{{(2\pi )}^{D}}{\int }_{0}^{{k}_{F}}dk\cdot {\alpha }_{D}{k}^{D-1}}=\frac{2{\pi }^{2}D}{D+2}\cdot {(\frac{D}{{\alpha }_{D}})}^{2/D}\cdot {\rho }^{2/D}.$$

It is noted that the definition of KEDF *t*[*ρ*] ≡ *δT*[*ρ*]/*δρ*, and its value is directly proportional to *ρ*^2/*D*^. For instance, the KEDF *t*[*ρ*] = (2*π*^2^/*α*_2_)*ρ* in a 2-dimensional system. Since the data weighting is directly proportional to the amplitude of the data PDF, the information weighting (i.e., the significance) in a studying system can be sufficiently described in terms of the KEDF. Additionally, the factor *α*_*D*_ can be merged into the adaptive scaling factor of Eq. (8), thus it will be set to be 1 in the following analyses.

Moreover, the form of electron-electron interaction *E*_*ee*_[*ρ*] actually represents the pair-particle interactions. Thus, this interacting form ingeniously constructs the mathematical configuration of information similarity so that the similarity between data points can be measured by their owning assigned PDF values and feature distances between each other. At the moment, the information similarity between pair-data-points can be measured using the potential energy density functional (PEDF):

7 $$u[\rho ]=\frac{\delta {E}_{ee}}{\delta \rho }=\frac{1}{2}{\int }^{}\frac{\rho ({\boldsymbol{r}}^{\prime} )}{|{\boldsymbol{r}}^{\prime\prime} -{\boldsymbol{r}}^{\prime} |}{d}^{D}{\boldsymbol{r}}^{\prime} .$$

where ***r″*** and ***r′*** are the feature coordinates of observation and source, respectively. It does not mean that the proposed method has to be limited to use the Coulombic form in the algorithm. The selection of a potential form actually relies on the data configuration of system^37^. To be specific, Eq. (7) provides an avenue for the measure of information similarity to pairs. The information similarity becomes very strong when the data pair are close to each other in the feature space. Furthermore, the form of Eq. (7) also reveals that the PEDF at ***r′′*** is weighted by the data PDF *ρ* from ***r′*** with a relative distance |***r″*** − ***r′***|.

Eventually, by employing the technique of Lagrangian multiplier with a constraint $N={\int }^{}\rho ({\boldsymbol{r}}^{\prime} ){d}^{D}{\boldsymbol{r}}^{\prime}$, Hamiltonian and Lagrangian density functionals (HDF and LDF) in a system of interest under the scheme of density functional theory can be respectively expressed as ${\mathcal H} [\rho ]={\gamma }^{2}t[\rho ]+\gamma u[\rho ]$ and ${\mathcal L} [\rho ]={\gamma }^{2}t[\rho ]-\gamma u[\rho ]$. The adaptive scaling factor *γ* can guarantee scale-free executions in the studying system and was derived from the differential of global Lagrangian with respect to the adaptive scaling factor: $\frac{d}{d\gamma }\,{\int }_{{\mathscr{P}}} {\mathcal L} [\rho ]\cdot \rho {d}^{D}{\boldsymbol{x}}^{\prime}$, the subscript ${\mathscr{P}}$ represents the operation space. Thus, by considering the non-trivial solution, we obtained

8 $$\gamma =\frac{1}{2}\frac{{\int }^{}u[\rho ]\cdot \rho {d}^{D}{\boldsymbol{r}}^{\prime} }{{\int }^{}t[\rho ]\cdot \rho {d}^{D}{\boldsymbol{r}}^{\prime} }=\frac{1}{2}\frac{\langle u[\rho ]\rangle }{\langle t[\rho ]\rangle }.$$

Consequently, the adaptive scaling factor is simply the ratio of global expectations between the PEDF and the KEDF. It is noted that γ is an adaptive factor. It can be uniquely and automatically determined by the properties of studying system.

## Results

**Figure 1:**
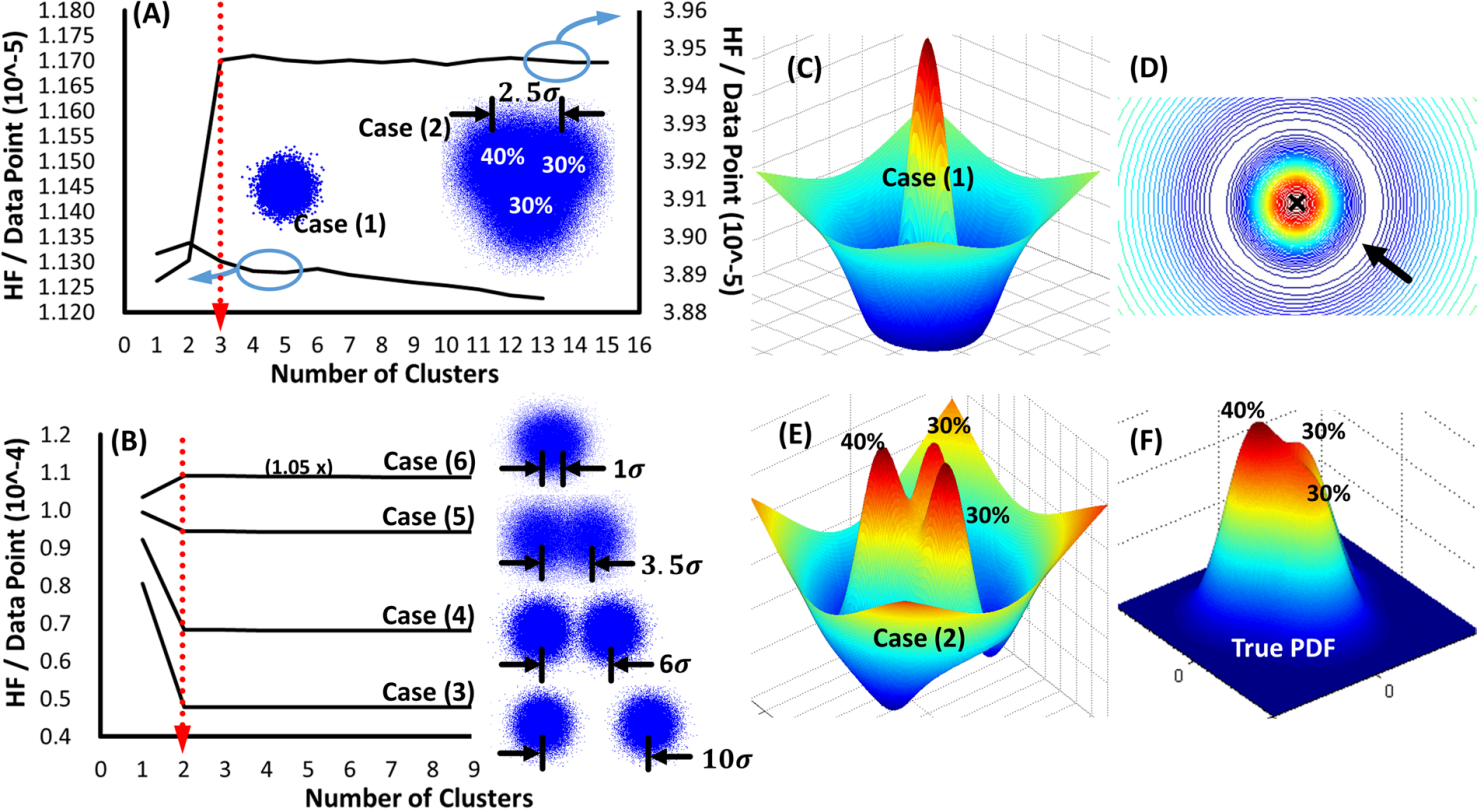
(**A**) and (**B**) show the results of unsupervised searching process of cluster number in various cases. The red dotted lines in (**A**) and (**B**) were used to indicate the estimated cluster number. The blue circles were used to demonstrate the data points of cluster distributions sampled by GMM. Only one cluster was employed in Case (1), while there were three clusters with different values of relative magnitude, 30%, 30% and 40%, in Case (2). The corresponding LDF landscapes of Case (1) and (2) are respectively illustrated as (**C**) and (**E**). The axes X and Y from (**C**) to (**E**) represent the feature axes in the data space, and the axis Z represents the estimated LDF in the corresponding coordinates. The axes X and Y in (**F**) have the same definition as in (**C**), but its axis Z represents the PDF magnitude. (**B**) shows the process of cluster number searching and the amalgamation of the clusters (illustrated from Case (3) to (6)). The regions delineated by the black arrows are the corresponding distances between the cluster centers. These distances were measured with the standard deviations σ of the sampled datasets. The white circular region indicated by the arrow in (**D**) delineates the cluster boundary of Case (1). The cross sample shown in the cluster center in (**D**) was estimated by k-means algorithm and used to confirm the position of cluster center.

The following analysis exhibits a route to deliver *the most probable PDF*, *the most probable cluster number*, and the corresponding *boundaries* in an arbitrary system. The common Gaussian mixture model (GMM)^52^ associated with the expectation-maximization (EM) algorithm^53^, both the popular and sophisticated methods in the field of machine learning, were used to generate idealized clusters and to construct global PDFs in this fictitious system. Figure 1 visually illustrates the unsupervised searching processes to the estimates of cluster numbers by averaging 85 GMM trials per round. In the case, the vector ***x***_i_ represents the coordinates of each data point, and the coefficient matrix *w* was obtained by learning from the datasets. For easy programming implementation, the 2-dimensional PEDF was simplified as $u[\rho ]\cong \sum _{n=1}^{N}\rho ({{\boldsymbol{r}}}_{n}\text{'}){\rm{\Delta }}{\boldsymbol{r}}/{|{\boldsymbol{r}}^{\prime\prime} -{{\boldsymbol{r}}}_{n}\text{'}|}_{{\boldsymbol{r}}^{\prime\prime} \ne {{\boldsymbol{r}}}_{n}\text{'}}$, where *n* is the location index of *n*th source point and the element Δ***r*** will be merged into Eq. (8) as *α*_*D*_ in the following study. ${{\bf{r}}}_{{\rm{n}}}\text{'}$s were sampled from the GMM and the corresponding data point distributions were also shown as the blue circle clusters in Fig. 1. The regions delineated by the black arrows are the corresponding distance between the cluster centers. These distances were measured using the standard deviations σ of the sampled datasets. The averaged Hamiltonian functional, ${\mathcal H}  {\mathcal F} =\{{\gamma }^{2}\langle t[\rho ]\rangle +\gamma \langle u[\rho ]\rangle \}/N$, was derived and then used to determine the cluster numbers as shown in Fig. 1(A) and (B).

### Case (1)

and (2) illustrated in Fig. 1(A) respectively represent a single uncharged atomic cluster and mixed atomic clusters with different relative amplitudes. The ${\mathcal H}  {\mathcal F}$ curve of Case (1) gradually decreases as the index of cluster number increasing, whereas the ${\mathcal H}  {\mathcal F}$ curve of Case (2) becomes stable after reaching a quasi-stationary point where the value is correspondingly equal to the most probable cluster number. The estimated cluster numbers in each case were marked up by the red-dotted arrows. The well-predicted value of the cluster number indicates that the most probable PDF were successfully estimated by the GMM method accordingly. The decreased ${\mathcal H}  {\mathcal F}$ curve in Case (1) can be attributed to missing the specific quasi-stationary point thus the estimation cannot reach the stationary state. Otherwise, the increased ${\mathcal H}  {\mathcal F}$ curve in Case (2) before reaching the quasi-stationary point reveals that more ${\mathcal H}  {\mathcal F}$ has been acquired to maintain the current configuration. Thus, this system tends to separate itself into subsystems to reduce the ${\mathcal H}  {\mathcal F}$ accumulation. After reaching the quasi-stationary point the ${\mathcal H}  {\mathcal F}$ curve becomes stable. Fluctuations after the quasi-stationary point were caused by the random noise from the GMM method.

In Fig. 1(B), the Case (3) to (6) were used to imitate the amalgamation of the uncharged atomic clusters, with peak-to-peak distances sequentially shrinking from 10 *σ* to *σ*. The quasi-stationary points were correctly estimated in every case as indicating by the red dotted arrow. Additionally, the gradually decreasing slopes of ${\mathcal H}  {\mathcal F}$ curves from Case (3) to (5) before arriving the quasi-stationary points reveal that the acquired ${\mathcal H}  {\mathcal F}$ increased while the clusters gradually approached with each other. Finally, the ${\mathcal H}  {\mathcal F}$ slope in Case (6) changes its direction due to the severely mixed clusters would like to separate spontaneously.

The morphologies shown in Fig. 1(C) and (E) are the LDF landscapes of Case 1 and 2 respectively, and the most probable boundaries of the clusters were definitely delineated. The minimum iso-surface shown in Fig. 1(D) clearly illustrates the unique cluster boundary by searching the zero points of ${\rm{\delta }} {\mathcal L} [\rho ]/\delta \rho$ as indicated by the black arrow. In Fig. 1(E), the most possible boundary of each cluster can be sequentially searched by finding the minimum ${\mathcal L} [\rho ]$ contour that contains only one cluster center since the cluster number has been given from the ${\mathcal H}  {\mathcal F}$ curve. Meanwhile, the shape of true PDF in Fig. 1(F) became steeper after mapping the clusters into the LDF scope as shown in Fig. 1(E) due to the limited activities of information communication. Thus, the severe mixed clusters become distinguishable.

In summary, the proposed scheme based on the density function theory provides an unsupervised learning method for non-parametrically determining the cluster number and the corresponding boundaries for a system of interest simultaneously. The plausibility of finding the most probable density by means of the proposed ${\mathcal H}  {\mathcal F}$ curve is erected on the quasi-stationary point searching. For elaborating the feasibility in realistic applications, the proposed scheme will also be applied to the pattern recognitions of different types of biological structures.

**Figure 2:**
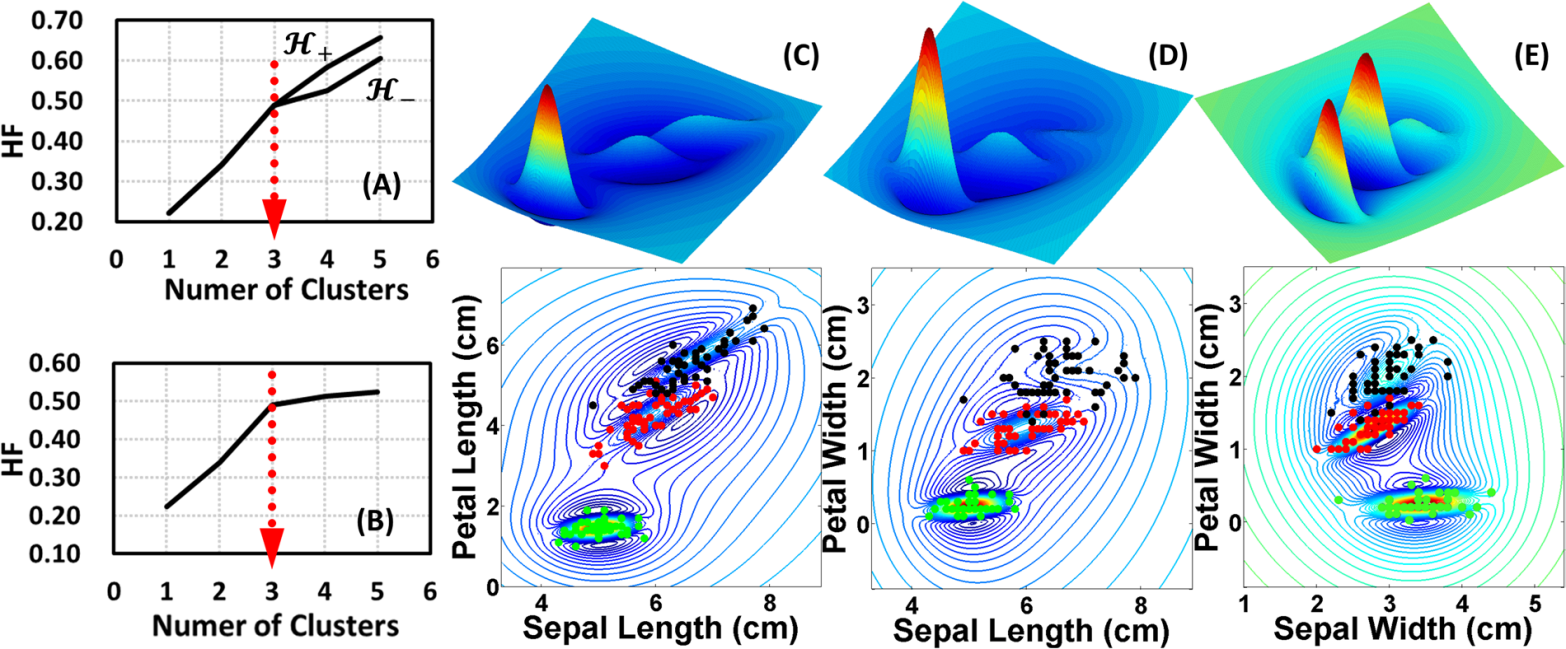
(**A**) and (**B**) respectively show the results of unsupervised searching and supervised intervention of possible cluster numbers from the Fisher’s iris dataset. The red dotted arrows in both ${\mathcal H}  {\mathcal F}$ curves indicate consistent searching results of cluster numbers to validate the proposed algorithm. Two ${\mathcal H}  {\mathcal F}$ branches in (**A**), ${{\boldsymbol{ {\mathcal H} }}}_{+}$ and ${{\boldsymbol{ {\mathcal H} }}}_{-}$, imply that there should be severely mixed clusters existed in the dataset. (**C**) to (**E**) sequentially illustrate the clustering results from the LDF landscapes with different feature axes. The series in the upper row exhibit the relative magnitudes of LDF landscapes, while the series of second row illustrate the LDF contours with the corresponding data points of clusters.

A classical problem in the pattern recognition, the Fisher’s iris, was employed from UC Irvine Machine Learning Repository^54^. There are three clusters of iris in the dataset, each cluster has four corresponding attributes, and each attribute includes 50 observations. For the method validation, only the information about attributes and corresponding observations were used. Statistically, resembling in the concept of ground state ensemble^55^ of density functional theory, the individual PDF of each cluster can be referred to Eq. (3), and the weighting factors between these PDFs *π*_*j*_ and their means ${{\boldsymbol{w}}}_{j}^{T}{{\boldsymbol{x}}}_{ij}$ were automatically learned by the GMM method associated with the EM algorithm. The estimation of 4-dimensional ${\mathcal H}  {\mathcal F}$ curve pertaining to the four attributes is shown as Fig. 2(A) and the quasi-stationary point occurred at the index of 3 as expected. The unsupervised searching process was truncated due to the ill-conditioned GMM estimates. To validate the searching results, the estimation with a supervised intervention is also shown in Fig. 2(B). The series in the upper row from Fig. 2(C) to (E) exhibit the relative magnitudes of LDF landscapes, while the series of second row illustrate the LDF contours with the corresponding data points of clusters. A significant phenomenon should be emphasized that the ${\mathcal H}  {\mathcal F}$ curve split at the quasi-stationary point and evolved into two branches, ${{\boldsymbol{ {\mathcal H} }}}_{+}$ and ${{\boldsymbol{ {\mathcal H} }}}_{-}$. As shown in the clustering results of the LDF landscapes from Fig. 2(C) to (E), two of the clusters were severely mixed with each other. Once these two mixed clusters were considered as one large cluster and the remaining one as a tiny one in the GMM estimates, their ${\mathcal H}  {\mathcal F}$ would stay at ${{\boldsymbol{ {\mathcal H} }}}_{+}$ state due to the large cluster had higher ${\mathcal H}  {\mathcal F}$. Otherwise, the ${\mathcal H}  {\mathcal F}$ would stay at ${{\boldsymbol{ {\mathcal H} }}}_{-}$ state once the clusters were considered as three roughly equal clusters. Thus, it is the reason why there was a ${\mathcal H}  {\mathcal F}$ split and it just occurred at the quasi-stationary point. Additionally, a non-ideal effect appears in the ${\mathcal H}  {\mathcal F}$ curve. Both ${{\boldsymbol{ {\mathcal H} }}}_{+}$ and ${{\boldsymbol{ {\mathcal H} }}}_{-}$ didn’t reach at stable states. It can be attributed to that the GMM method cannot offer exact PDFs and consequently the searches cannot be terminated. It is plausible that the predicament can be conquered by further linking the method of Bayesian sequential partitioning^56^ in the future.

**Figure 3:**
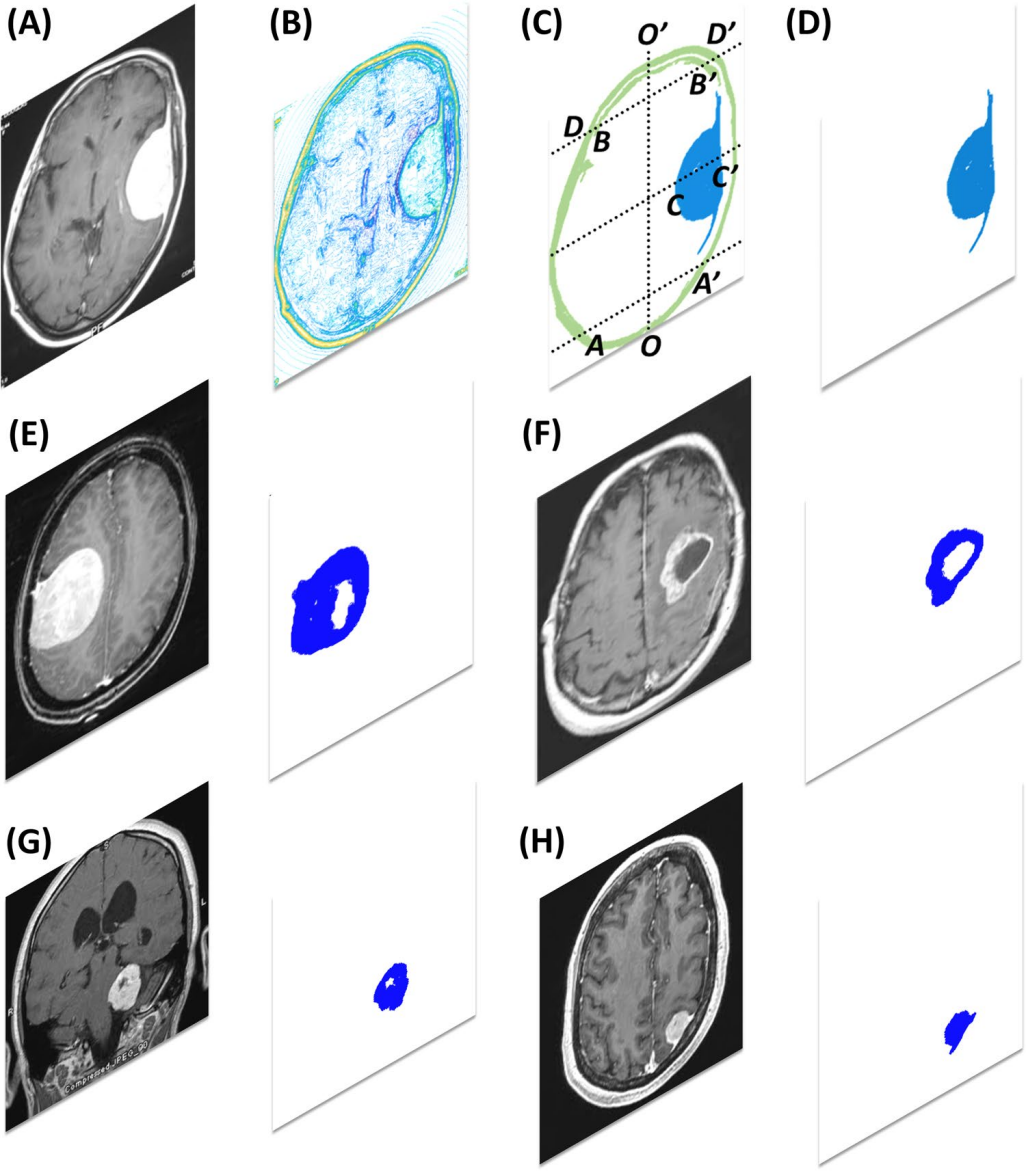
The original MRI datasets were sourced from the open data in ref.^57^. The width and height of the original image of (**A**) are 205 and 246 pixels, respectively. (**B**) and (**C**) show the LDF contour and its segmentation, respectively. The undesired components in (**C**) can be removed using their quasi-symmetrical characteristics. The components that included $\overline{AA\text{'}}$, $\overline{BB\text{'}}$, and $\overline{DD\text{'}}$ were separated by $\overline{OO\text{'}}$, so these components were removed from the candidate dataset of segmentation. The final segmentation is shown in (**D**). From (**E**) to (**H**), the results of segmentation of brain tumors are also exhibited aside.

Then, the brain tumor detections of magnetic resonance imaging (MRI) datasets and the segmentation of a neural network in a *Brainbow* system were analyzed. In the case of MRI, a one-dimensional pixel intensity distribution embedded in a 2-dimensional image frame was adopted to construct the intensity PDF, $\rho ({\boldsymbol{r}}^{\prime} )=\sum _{n=1}^{W\times H}{M}_{n}\times \delta ({\boldsymbol{r}}^{\prime} -{{\boldsymbol{r}}}_{n}\text{'})$, where *W* and *H* are respectively the width and height of images, ${{\boldsymbol{r}}}_{n}\text{'}$ is now the position of the *n*th pixel, and *M*_*n*_ is the corresponding normalized intensity. Thus, the 3-dimensional information was reduced into a 1-dimensional intensity PDF. For the convenience of programming, the PEDF was simplified as $u[\rho ]\cong \sum _{n=1}^{W\times H}{M}_{n}/{|{\boldsymbol{r}}^{\prime\prime} -{{\boldsymbol{r}}}_{n}\text{'}|}_{{{\boldsymbol{r}}}^{^{\prime\prime} }\ne {{\boldsymbol{r}}}_{n}\text{'}}$. Figure 3 shows the results of tumor segmentation, where the original MRI datasets were sourced from ref.^57^. In the LDF contour of Fig. 3(B), the normal tissues and the tumor have distinguishable co-edges. However, these tissues also have distinguishable co-edges between their interfaces, such as the interfaces between white and gray matters and that between soft tissues and skull. The automated image segmentation from the LDF contour is shown in Fig. 3(C). As expected, not only the tumor image was extracted but also other undesired components were also detected and segmented. These undesired components were further automatically removed using the quasi-symmetrical configuration of the original imagery. The components shown in Fig. 3(C) that included $\overline{AA\text{'}}$, $\overline{BB\text{'}}$, and $\overline{DD\text{'}}$ were separated by $\overline{OO\text{'}}$, so these components were removed from the candidate dataset of segmentation. The final segmentation is as shown in Fig. 3(D). To validate the proposed unsupervised learning algorithm, four other MRI datasets sourced from ref.^57^ were employed and analyzed as shown from Fig. 3(E) to (H). The results of tumor segmentations confirm the feasibility of the proposed algorithm.

**Figure 4:**
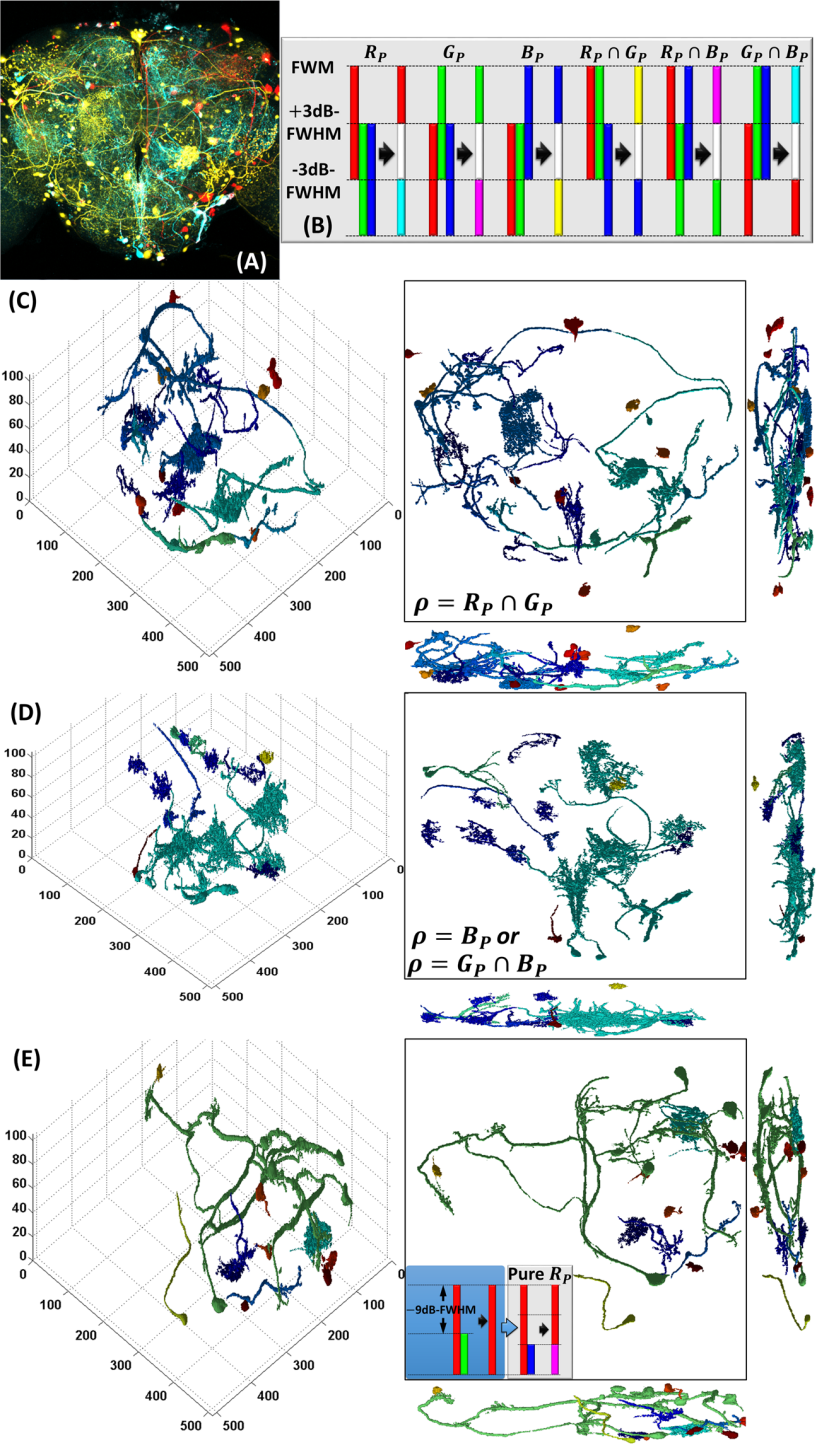
The original *Brainbow* imagery and the procedure of ±3*n*dB-FWHM (*n* = 1) are all shown in (**A**) and (**B**), respectively. Each image slice spanned in a 1024 × 1024 pixel space and the whole system collected 105 slices in depth. Each physical volume correspondingly occupies 354 μm × 354 μm × 105 μm. In order to reduce the computational complexity and filtrate out the background noises, the corresponding 3-dimensional *Brainbow* images were processed slice-by-slice by the wavelet technique in depth. The results of segmentation and their corresponding color intensity PDF are shown from (**C**) to (**E**). Two of the coordinate scales were shrunk with 50% due to the level 1 wavelet transform. Results sequentially shown in (**C**) to (**E**) are the coloring neural networks in yellow, cyan, and red channels, respectively. Different coloring parts in each results exhibit the different clusters in the corresponding color channels. Each figure shows the three-dimensional morphology and the projections in each direction of axis under specific HDF estimation. Due to the pure red information is embedded in the yellow channel, an additional filtering mechanism with *n* = 3 was adopted for the pure red channel extraction and as shown in (**E**).

The *Brainbow* dataset is an assembly of 3-dimensional color intensity distribution that spans in a large-scale 3-dimensional pixel space. In the case, the employed 6-dimensional *Brainbow* imagery was produced at the Brain Research Center in National Tsing Hua University, Taiwan^25^ and the corresponding projection is shown in Fig. 4(A). Each image slice spanned in a 1024 × 1024 pixel space and the whole system collected 105 slices in depth. Since neural morphologies usually suffered severe color crosstalks then result in the degradation of discrimination ability of state-of-the-art techniques as mentioned in Introduction Section, a technique of ±3*n*dB-FWHM (full width at half maximum) was introduced as a pre-processing filter, where the factor *n* was employed to describe the systematic complexity. A similar filtering technique can be referred to ref.^58^. In the technique, one of each *RGB* color channel was sequentially employed as a principal channel while the remaining channels became auxiliary ones. For instance, if red channel *R* is adopted as the principal one *R*_*P*_ for *n* = 1, we have:

9 $$\begin{array}{ccc}R\equiv {R}_{P} & \equiv  & \{\begin{array}{cc}1, & {\rm{intensity}}\ge -3{\rm{dB}}-{\rm{FWHM}}\\ 0, & {\rm{otherwise}}\end{array}\\ G & = & \{\begin{array}{cc}1, & {\rm{intensity}}\le +3{\rm{dB}}-{\rm{FWHM}}\\ 0, & {\rm{otherwise}}\end{array}\\ B & = & \{\begin{array}{cc}1, & {\rm{intensity}}\le +3{\rm{dB}}-{\rm{FWHM}}\\ 0, & {\rm{otherwise}}\end{array}\end{array}$$

Thus the filtered PDF becomes $\rho ={R}_{P}\cap G\cap B$ for *n* = 1 in the case as shown in the first column of Fig. 4(B). These three distinct color channels were mixed to construct a specific dimension-reduced channel: pure red channel upon the +3dB-FWHM, mixtures with all channels bounded by ±3dB-FWHM, and mixtures with green and blue below the −3dB-FWHM. In other words, the 3-dimensional intensity PDF can simultaneously carry all of these information about the mixed channels and the corresponding coordinates. The remaining color blending mechanisms are illustrated in Fig. 4(B) for the case of *n* = 1. There are 2^3^ − 2 routes of the color mixing mechanism, excluded the cases of no principal channel and all colors participating in the principal channels. Namely, for the purpose of automatic feature selection and complexity reduction in the case, only 6 possible options of mixed-color intensity PDF and 3 options of pure-color intensity PDF should be sequentially considered in the *Brainbow* system.

After the pre-processing filtering, the corresponding 3-dimensional *Brainbow* images were processed slice-by-slice by the wavelet technique in depth. In order to reduce the computational complexity and filtrate out the background noises, level 1 wavelet transform and Haar wavelet filter are respectively used for further data compression and denoising. Eventually, all of the processed slices were then sequentially collected to constitute a 3-dimensional filtered dataset. To segregate potential neurons, whole dataset was digitized as the procedure listed in Eq. (9). The PEDF estimate of each voxel only used 26-connected neighboring components^20^. Then the corresponding neurons could be morphologically recognized by finding the minimum iso-surfaces of HDF. The derived HDF morphologies are shown from Fig. 4(C) to (D). Each figure shows the 3-dimensional HDF landscapes and the corresponding morphological projections in each direction of pixel axis. Two of the coordinate scales were shrunk with 50% due to the level 1 wavelet transform. By comparing the projection of Fig. 4(A), these results sequentially were the coloring neural networks classified in yellow, cyan, and red channels, respectively. Different coloring parts in each figure exhibit the different clusters in the corresponding channels.

It is found that the basic red channel was mixed with the other combinatorial channels so that their corresponding neural morphologies would be mingled severely. Thus, the red-channel-based neural morphology would not be definitely presented unless the auxiliary channels could be first inhibited. As shown in Fig. 4(E), a threshold value of −9dB-FWHM (*n* = 3) in the case was set to inhibit the performance from green signals. Then the filtered red intensity and the rest blue one were used to construct the localized intensity PDF, $\rho ={R}_{P}{\cap }^{}B$, for the further HDF estimation.

## Discussion

In conclusion, a compact method for unsupervised learning and pattern recognitions based on the density functional theory and the machine learning methods has been successfully applied to interdisciplinary problems, providing informative findings based on physical intuition. In the case of the amalgamation of the uncharged atomic clusters in a 2-dimensional physical space, the most probable cluster number and the corresponding cluster boundaries can be respectively determined by the indication of the quasi-stationary point occurring on the ${\mathcal H}  {\mathcal F}$ curve and the LDF landscape simultaneously. The PDF estimate might use the GMM method associated with the EM algorithm. The proposed unsupervised searching method can be theoretically extended to high-dimensional data space, and this has been validated by the 4-dimensional Fisher’s iris datasets. Especially, the ${\mathcal H}  {\mathcal F}$ split happens once the clusters are severely mixed.

The connection between density functional theory and machine learning methods leads to the new perspective for unsupervised pattern recognitions. The computational complexity reduction of PDF estimates can be achieved by introducing the mathematical framework of the density functional theory to the data systems. The morphologies of LDF reveal significant data boundaries between clusters, while that of HDF connects the components having the most similar local information. Furthermore, in the systematic applications, the proposed unsupervised learning algorithm can be combined with other techniques from the contemporary machine learning methods. For instance, the concept of connected components from graph theory was employed to reduce the computational complexity of functional calculations. Moreover, the wavelet technique was used for data compression and denoising. The brain tumor detections from low-dimensional MRI datasets and the segmentations of high-dimensional *Brainbow* system were used to evaluate the method in practice. In the study of brain tumor detections of MRI datasets, the key information related to the physical locations, shapes, and sizes of the detected tumors can be extracted by tracing the relevant pixel matrices using the proposed algorithm. Meanwhile, the interfaces between the surrounding tissues and the candidate tumors can be delineated by finding the locations of LDF mismatches. In addition, in the study of neural segmentation of the *Brainbow* system, the 3-dimensional configuration of the intricate neural networks was also detected using the proposed algorithm. Thus, the key information obtained by this systematic approach can provide useful suggestion to the relevant investigators to track specific neural circuits under any specific drug stimulation or external stress. The experimental results also exhibit the successful connection between the physical theory and the machine learning methods.

Therefore, the proposed method successfully integrates the merits from the physical methods and the popular machine learning methods. To make contribution to the clinical investigation by translational science, the desired technique should extract the key information by means of objective methods for pattern recognition and automatic segmentation. In addition to solving the demanding segmentation and recognition in high-dimensional biomedical problems, the proposed scheme can be further implemented to the major topics in science, such as the computer vision, connectomics, DIADEM (digital reconstruction of axial and dendritic morphology) challenges, and so forth.

## Electronic supplementary material

README

DDFT_MRI_AllinOne

DDFT_MRI_EnergyCalculationPlot

Supplementary figures
