# Supplementary material for: Unsupervised Learning and Pattern Recognition of Biological Data Structures with Density Functional Theory and Machine Learning
Source: Sci Rep. 2018 Jan 11;8:557. doi: 10.1038/s41598-017-18931-5 (PMC5765025; doi:10.1038/s41598-017-18931-5)
Supplement: Supplementary file 1 — README [file 41598_2017_18931_MOESM1_ESM.pdf]

# Unsupervised Learning and Pattern Recognition of Biological Data Structures with Density Functional Theory and Machine Learning

Chien-Chang Chen,[1,2] Hung-Hui Juan,[2] Meng-Yuan Tsai,[3] and Henry Horng-Shing Lu[2,3,4,*]

[1]Bio-Microsystems Integration Laboratory, Department of Biomedical Sciences and Engineering, National Central University, Taoyuan City, Taiwan

[2]Shing-Tung Yau Center, National Chiao Tung University, 1001 University Road, Hsinchu City, Taiwan

[3]Institute of Statistics, National Chiao Tung University, 1001 University Road, Hsinchu City, Taiwan

[4]Big Data Research Center, National Chiao Tung University, 1001 University Road, Hsinchu City, Taiwan

* hslu@stat.nctu.edu.tw

VERSION of this file : Oct. 29th, 2017

Note:

(1) The codes were built on MATLAB 2015b.

(2) Function of parallel calculation was used in the part of "(3) KEDF & PEDF setting and calculations"
in "DDFT_MRI_AllinOne.m" file.

Default core number is 12, and has been used in line 22 and 35:

```
p = parpool('local', 12);    % Open the function
delete(p);                   % Close the function
```

Please modify this first according to your personal computer conditions.

Start:

(1) Open "DDFT_MRI_AllinOne.m" in MATLAB environment.
Modify the file name in Line 3.
Default is:

```
file_name = '1Perfect.jpg';
```

Five files can be employed: 1Perfect.jpg, 2Perfect.jpg, 5Perfect.jpg, 6Perfect.jpg, and 6Perfect2.jpg.

(2) Run "DDFT_MRI_AllinOne.m"

(2-1) PDF of MRI will be initialized and set in step (1) and (2).
(2-2) KEDF and PEDF will be estimated in step (3).
(2-3) HDF and LDF will then be estimated in step (4).
(2-4) All results will be exhibited also in step (4) using "DDFT_MRI_EnergyCalculationPlot.m"

Results:

(1) Original MRI will be shown in Figure (1).
All status underwent will be listed below the figure.

(2) Distributions and Coutours of KEDF, PEDF, HDF, and LDF will be respectively shown in following figure windows.

(3) Segmentation result will be marked on the original MRI and also shown in Figure (1).
The execution time will be also listed.
