# Supplementary material for: Unsupervised Learning and Pattern Recognition of Biological Data Structures with Density Functional Theory and Machine Learning
Source: Sci Rep. 2018 Jan 11;8:557. doi: 10.1038/s41598-017-18931-5 (PMC5765025; doi:10.1038/s41598-017-18931-5)
Supplement: Supplementary file 2 — DDFT_MRI_AllinOne [file 41598_2017_18931_MOESM2_ESM.pdf]

Unsupervised Learning and Pattern Recognition of Biological Data Structures with Density Functional Theory and Machine Learning

Chien-Chang Chen,[1,2] Hung-Hui Juan,[2] Meng-Yuan Tsai,[3] and Henry Horng-Shing Lu[2,3,4,*]

[1]Bio-Microsystems Integration Laboratory, Department of Biomedical Sciences and Engineering, National Central University, Taoyuan City, Taiwan

[2]Shing-Tung Yau Center, National Chiao Tung University, 1001 University Road, Hsinchu City, Taiwan

[3]Institute of Statistics, National Chiao Tung University, 1001 University Road, Hsinchu City, Taiwan

[4]Big Data Research Center, National Chiao Tung University, 1001 University Road, Hsinchu City, Taiwan

* hslu@stat.nctu.edu.tw

```matlab
%% File Name : DDFT_MRI_AllinOne
%% (1) initial conditions
   clear; close all;clc;
   file_name = '1Perfect.jpg';           % File name
   timem = tic;
%% (2) PDF setting
   I_ori = double(imread(file_name))/255;
   I_ori = I_ori.^2;
   [H,W,c] = size(I_ori);
   if c==3
      I_ori = rgb2gray(I_ori);    % RGB to gray level
   end
   I = I_ori/(sum(sum(I_ori)));    % normalization of PDF
%% (3) KEDF & PEDF setting and calculations
   [x y] = meshgrid(1:1:W,1:1:H);     %% Energy distribution's grids
   R0 = [x(:) y(:)]; Ivec = I(:);
% Original image
   figure(1); subplot(1,2,1), imshow(I_ori); title('Original Image');
% KEDF calculation
   first_term = 2*pi*Ivec; disp('KED done!'); text(0,1.05*H,'KEDF done!')
% PEDF calculaton
   text(0,1.15*H,'Starting Parallel Calculations.....Please Wait!')
   p = parpool('local', 12);
      parfor h = 1 : H
      for w = 1 : W
         VED_temp = zeros(H,W);
         VED_temp(h,w)=1;
         dist = bwdist(VED_temp,'euclidean');
         dist_inv = 1./dist;
         dist_inv(isinf(dist_inv))=0;
         potential_temp = 1/2*dist_inv.*reshape(Ivec,H,W);
         potential_sum(h,w)  = sum(sum(potential_temp));
      end
      end
   text(0,1.25*H,'PEDF done!')
   delete(p);   disp('VED done!');
   %delete(gcp('nocreate'))
%% (4) Energy Calculation
   V = potential_sum;
% gamma estimation
   M = length(I(:));
   mean_KE = sum(first_term.*I(:))/M;
```

```matlab
    mean_VE = sum(V(:).*I(:))/M;
    gamma = 0.5*mean_VE/mean_KE;

    KED = first_term * gamma^2;
    VD = V *gamma;
    VD = VD(:);
    HED = VD + KED;
    LED = -VD + KED;
% matrix reshapes
    KED = double(reshape(KED,H,W));
    VD = double(reshape(VD,H,W));
    HED = double(reshape(HED,H,W));
    LED = double(reshape(LED,H,W));
toc(timem);

DDFT_MRI_EnergyCalculationPlot;

disp('finished!!'); text(0.5*W,1.1*H,'Finished!!');
```
