# Supplementary material for: Unsupervised Learning and Pattern Recognition of Biological Data Structures with Density Functional Theory and Machine Learning
Source: Sci Rep. 2018 Jan 11;8:557. doi: 10.1038/s41598-017-18931-5 (PMC5765025; doi:10.1038/s41598-017-18931-5)
Supplement: Supplementary file 3 — DDFT_MRI_EnergyCalculationPlot [file 41598_2017_18931_MOESM3_ESM.pdf]

Unsupervised Learning and Pattern Recognition of Biological Data Structures with Density Functional Theory and Machine Learning

Chien-Chang Chen,[1,2] Hung-Hui Juan,[2] Meng-Yuan Tsai,[3] and Henry Horng-Shing Lu[2,3,4,*]

[1]Bio-Microsystems Integration Laboratory, Department of Biomedical Sciences and Engineering, National Central University, Taoyuan City, Taiwan

[2]Shing-Tung Yau Center, National Chiao Tung University, 1001 University Road, Hsinchu City, Taiwan

[3]Institute of Statistics, National Chiao Tung University, 1001 University Road, Hsinchu City, Taiwan

[4]Big Data Research Center, National Chiao Tung University, 1001 University Road, Hsinchu City, Taiwan

* hslu@stat.nctu.edu.tw

```matlab
%% DDFT_MRI_EnergyCalculationPlot
figure(2);
subplot(1,2,1);
h = surf(x, y, KED); set(h, 'EdgeAlpha', 0.05);
title('KEDF Distribution');
axis normal; axis ij;
subplot(1,2,2);
contour(x, y, KED, 50);
title('Contour of KEDF');  colorbar;
axis square;  axis ij;

figure(3);
subplot(1,2,1);
h = surf(x, y, VD); set(h, 'EdgeAlpha', 0.05);
title('PEDF Distribution');
axis normal; axis ij;
subplot(1,2,2);
contour(x, y, VD, 50);
title('Contour of PEDF');   colorbar;
axis square; axis ij;

figure(4);
subplot(1,2,1);
h = surf(x, y, HED); set(h, 'EdgeAlpha', 0.05);
title('HDF Distribution');
axis normal;    axis ij;
subplot(1,2,2);
contour(x, y, HED, 50);
title('Contour of HDF');    colorbar;
axis square;    axis ij;

figure(5);
subplot(1,2,1);
h = surf(x, y, LED); set(h, 'EdgeAlpha', 0.05);
title('LDF Distribution');
axis normal;    axis ij;
subplot(1,2,2);
contour(x, y, LED, 50);
title('Contour of LDF'); colorbar;
axis square;    axis ij;
```

```
LDF = (LED>=mean(LED(:)));
CC = bwconncomp(LDF);
numPixels = cellfun(@numel,CC.PixelIdxList);
[biggest,idx] = max(numPixels);
title_check = 1;

for con_idx = 1:4   % take the first three components
    indexMAX = CC.PixelIdxList{1,idx};
    [XX YY] = ind2sub(size(I_ori),indexMAX);

    if ~(min(XX)<H/8) & ~(min(YY)<W/8) & title_check==1
        figure(1), text(0,1.35*H,['Overall Time : ', num2str(toc(timem)), ' sec.'])
        subplot(1,2,2), imshow(I_ori); hold on
        scatter(YY,XX,'.','r'); axis ij, axis equal, axis([0 W 0 H]);
        title('Candidate'), title_check = 0;
    end

    CC.PixelIdxList{1,idx}=[];
    numPixels = cellfun(@numel,CC.PixelIdxList);
    [biggest,idx] = max(numPixels);
end
```
