# Supplementary material for: Unsupervised Learning and Pattern Recognition of Biological Data Structures with Density Functional Theory and Machine Learning
Source: Sci Rep. 2018 Jan 11;8:557. doi: 10.1038/s41598-017-18931-5 (PMC5765025; doi:10.1038/s41598-017-18931-5)
Supplement: Supplementary file 4 — Supplementary figures [file 41598_2017_18931_MOESM4_ESM.pdf]

# Unsupervised Learning and Pattern Recognition of Biological Data Structures with Density Functional Theory and Machine Learning

Chien-Chang Chen,[1,2] Hung-Hui Juan,[2] Meng-Yuan Tsai,[3] and Henry Horng-Shing Lu[2,3,4,*]

[1]Bio-Microsystems Integration Laboratory, Department of Biomedical Sciences and Engineering, National Central University, Taoyuan City, Taiwan

[2]Shing-Tung Yau Center, National Chiao Tung University, 1001 University Road, Hsinchu City, Taiwan

[3]Institute of Statistics, National Chiao Tung University, 1001 University Road, Hsinchu City, Taiwan

[4]Big Data Research Center, National Chiao Tung University, 1001 University Road, Hsinchu City, Taiwan

* hslu@stat.nctu.edu.tw

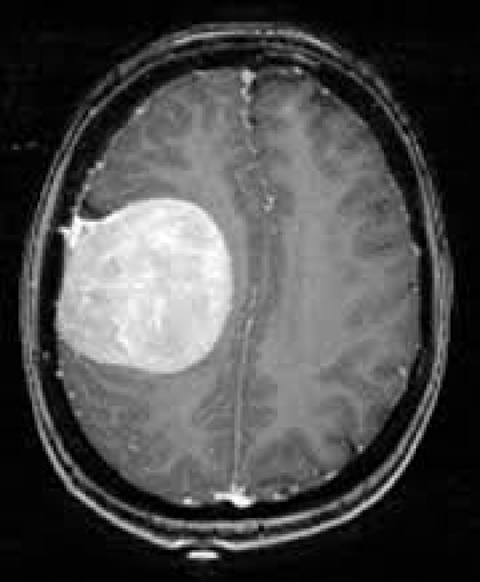

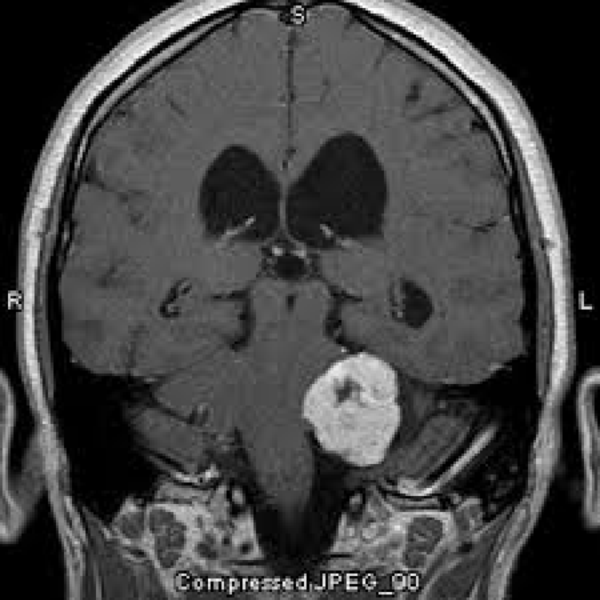
S
R
L
Compressed JPEG_90

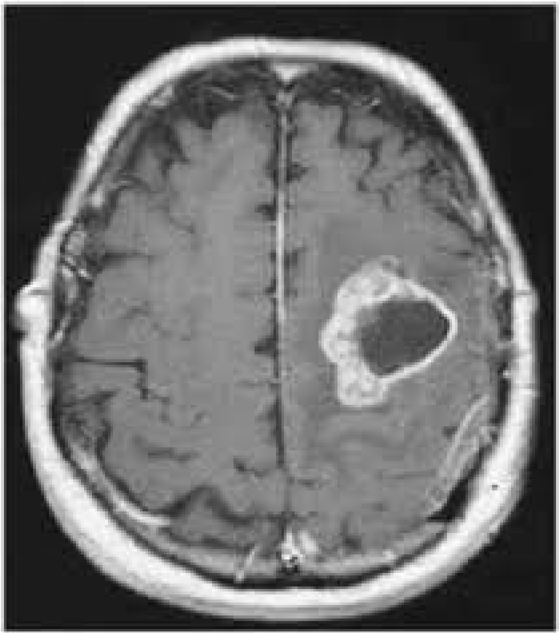

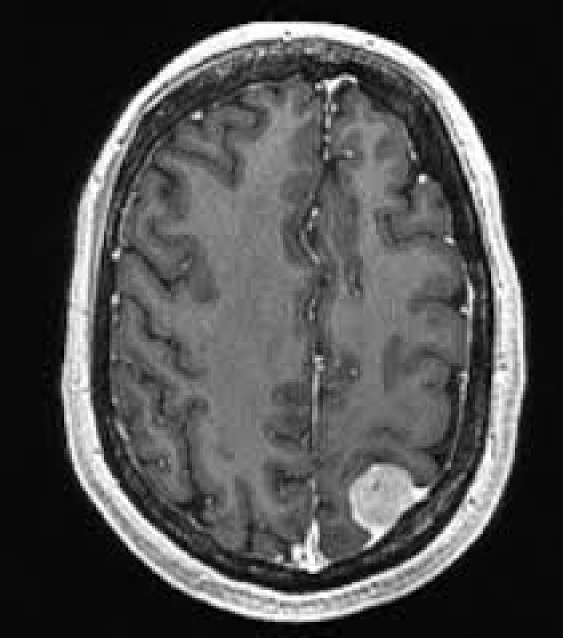

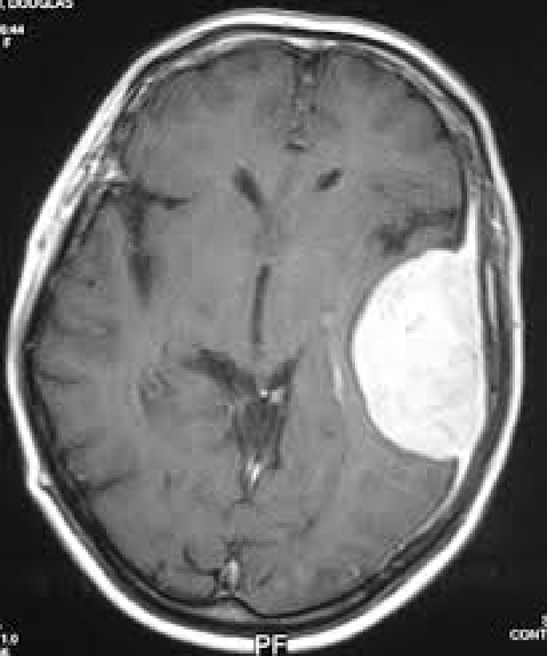
PF
